# Molecular Signatures of Major Depression

**DOI:** 10.1016/j.cub.2015.03.008

**Published:** 2015-05-04

**Authors:** Na Cai, Simon Chang, Yihan Li, Qibin Li, Jingchu Hu, Jieqin Liang, Li Song, Warren Kretzschmar, Xiangchao Gan, Jerome Nicod, Margarita Rivera, Hong Deng, Bo Du, Keqing Li, Wenhu Sang, Jingfang Gao, Shugui Gao, Baowei Ha, Hung-Yao Ho, Chunmei Hu, Jian Hu, Zhenfei Hu, Guoping Huang, Guoqing Jiang, Tao Jiang, Wei Jin, Gongying Li, Kan Li, Yi Li, Yingrui Li, Youhui Li, Yu-Ting Lin, Lanfen Liu, Tiebang Liu, Ying Liu, Yuan Liu, Yao Lu, Luxian Lv, Huaqing Meng, Puyi Qian, Hong Sang, Jianhua Shen, Jianguo Shi, Jing Sun, Ming Tao, Gang Wang, Guangbiao Wang, Jian Wang, Linmao Wang, Xueyi Wang, Xumei Wang, Huanming Yang, Lijun Yang, Ye Yin, Jinbei Zhang, Kerang Zhang, Ning Sun, Wei Zhang, Xiuqing Zhang, Zhen Zhang, Hui Zhong, Gerome Breen, Jun Wang, Jonathan Marchini, Yiping Chen, Qi Xu, Xun Xu, Richard Mott, Guo-Jen Huang, Kenneth Kendler, Jonathan Flint

**Affiliations:** 1 Wellcome Trust Centre for Human Genetics, University of Oxford, Roosevelt Drive, Oxford, Oxfordshire OX3 7BN, UK; 2 Department and Graduate Institute of Biomedical Sciences, College of Medicine, Chang Gung University, Tao-Yuan 33302, Taiwan, ROC; 3 BGI-Shenzhen, Floor 9 Complex Building, Beishan Industrial Zone, Yantian District, Shenzhen, Guangdong 518083, China; 4 Department of Comparative Developmental Genetics, Max Planck Institute for Plant Breeding Research, Carl-von-Linne-Weg 10, Cologne 50829, Germany; 5 Centro de Investigacion Medica en Red de Salud Mental, CIBERSAM-University of Granada, Granada, Spain; 6 MRC SGDP Centre, Institute of Psychiatry at King’s College, De Crespigny Park, London SE5 8AF, UK; 7 National Institute for Health Research, Biomedical Research Centre for Mental Health, Institute of Psychiatry at King’s College, De Crespigny Park, London SE5 8AF, UK; 8 Mental Health Center of West China Hospital of Sichuan University, No. 28 South Dianxin Street, Wuhou District, Chengdu, Sichuan 610000, China; 9 Hebei Mental Health Center, No. 572 Dongfeng Road, Baoding, Hebei 71000, China; 10 Zhejiang Traditional Chinese Medical Hospital, No. 54 Youdian Road, Hangzhou, Zhejiang 310000, China; 11 Ningbo Kang Ning Hospital, No. 1 Zhuangyu Road, Zhenhai District, Ningbo, Zhejiang 315000, China; 12 Liaocheng No. 4 Hospital, No. 47 North Huayuan Road, Liaocheng, Shandong 252000, China; 13 Department of Medical Biotechnology and Laboratory Science, College of Medicine, Chang Gung University, Tao-Yuan 33302, Taiwan, ROC; 14 No. 3 Hospital of Heilongjiang Province, No. 135 Jiaotong Road, Beian, Heilongjiang 164000, China; 15 Harbin Medical University, No. 23 Youzheng Street, Nangang District, Haerbin, Heilongjiang 150000, China; 16 Sichuan Mental Health Center, No. 190, East Jiannan Road, Mianyang, Sichuan 621000, China; 17 Chongqing Mental Health Center, No. 102 Jinzishan, Jiangbei District, Chongqing, Chongqing 404100, China; 18 Mental Health Institute of Jining Medical College, Dai Zhuang, Bei Jiao, Jining, Shandong 272000, China; 19 Mental Hospital of Jiangxi Province, No. 43 Shangfang Road, Nanchang, Jiangxi 330000, China; 20 Wuhan Mental Health Center, No. 70, Youyi Road, Wuhan, Hubei 430000, China; 21 No. 1 Hospital of Zhengzhou University, No. 1 East Jianshe Road, Zhengzhou, Henan 450000, China; 22 Graduate Institute of Biomedical Sciences, College of Medicine, Chang Gung University, Tao-Yuan 33302, Taiwan, ROC; 23 Shandong Mental Health Center, No. 49 East Wenhua Road, Jinan, Shandong 250000, China; 24 Shenzhen Key Lab for Psychological Healthcare, Kangning Hospital, No. 1080, Cuizhu Street, Luohu District, Shenzhen, Guangdong 518000, China; 25 The First Hospital of China Medical University, No. 155 North Nanjing Street, Heping District, Shenyang, Liaoning 110001, China; 26 Psychiatric Hospital of Henan Province, No. 388 Middle Jianshe Road, Xinxiang, Henan 453000, China; 27 No. 1 Hospital of Chongqing Medical University, No. 1 Youyi Road, Yuanjiagang, Yuzhong District, Chongqing, Chongqing 400016, China; 28 Changchun Mental Hospital, No. 4596 Beihuan Road, Changchun, Jilin 130000, China; 29 Tianjin Anding Hospital, No. 13 Liulin Road, Hexi District, Tianjin, Tianjin 300000, China; 30 Xian Mental Health Center, No. 15 Yanyin Road, New Qujiang District, Xian, Shaanxi 710000, China; 31 Brain Hospital of Nanjing Medical University, No. 264 Guangzhou Road, Nanjing, Jiangsu 210000, China; 32 Second Affiliated Hospital of Zhejiang Chinese Medical University, No. 318 Chaowang Road, Hangzhou, Zhejiang 310000, China; 33 Beijing Anding Hospital of Capital University of Medical Sciences, No. 5 Ankang Hutong, Deshengmen wai, Xicheng District, Beijing, Beijing 100000, China; 34 First Hospital of Hebei Medical University, No. 89 Donggang Road, Shijiazhuang, Hebei 50000, China; 35 ShengJing Hospital of China Medical University, No. 36 Sanhao Street, Heping District, Shenyang, Liaoning 110001, China; 36 Jilin Brain Hospital, No. 98 West Zhongyang Road, Siping, Jilin 136000, China; 37 No. 3 Hospital of Sun Yat-sen University, No. 600 Tianhe Road, Tianhe District, Guangzhou, Guangdong 510000, China; 38 No. 1 Hospital of Shanxi Medical University, No. 85 South Jiefang Road, Taiyuan, Shanxi 30000, China; 39 Daqing No. 3 Hospital of Heilongjiang Province, No. 54 Xitai Road, Ranghulu District, Daqing, Heilongjiang 163000, China; 40 No. 4 Hospital of Jiangsu University, No. 246 Nanmen Street, Zhenjiang, Jiangsu 212000, China; 41 Anhui Mental Health Center, No. 316 Huangshan Road, Hefei, Anhui 230000, China; 42 Department of Biology, University of Copenhagen, Ole Maaloes Vej 5, Copenhagen 2200, Denmark; 43 Macau University of Science and Technology, Avenida Wai Long, Taipa, Macau 999078, China; 44 Princess Al Jawhara Center of Excellence in the Research of Hereditary Disorders, King Abdulaziz University, Jeddah 21589, Saudi Arabia; 45 Department of Statistics, University of Oxford, Oxford, Oxfordshire OX1 3TG, UK; 46 CTSU, University of Oxford, Richard Doll Building, Old Road Campus, Oxford, Oxfordshire OX3 7LF, UK; 47 National Laboratory of Medical Molecular Biology, Institute of Basic Medical Sciences & Neuroscience Center, Chinese Academy of Medical Sciences and Peking Union Medical College, Beijing 10005, China; 48 Dept Psychiatry MCV, Virginia Commonwealth University, Richmond, VA 23298, USA; 49 East China Normal University, 3663 North Zhongshan Road, Shanghai 200062, China

## Abstract

Adversity, particularly in early life, can cause illness. Clues to the responsible mechanisms may lie with the discovery of molecular signatures of stress, some of which include alterations to an individual’s somatic genome. Here, using genome sequences from 11,670 women, we observed a highly significant association between a stress-related disease, major depression, and the amount of mtDNA (p = 9.00 × 10^−42^, odds ratio 1.33 [95% confidence interval [CI] = 1.29–1.37]) and telomere length (p = 2.84 × 10^−14^, odds ratio 0.85 [95% CI = 0.81–0.89]). While both telomere length and mtDNA amount were associated with adverse life events, conditional regression analyses showed the molecular changes were contingent on the depressed state. We tested this hypothesis with experiments in mice, demonstrating that stress causes both molecular changes, which are partly reversible and can be elicited by the administration of corticosterone. Together, these results demonstrate that changes in the amount of mtDNA and telomere length are consequences of stress and entering a depressed state. These findings identify increased amounts of mtDNA as a molecular marker of MD and have important implications for understanding how stress causes the disease.

## Highlights

• Amount of mtDNA is increased, and telomeric DNA is shortened in major depression
• Both changes can be induced with stress but are contingent on the depressed state
• Changes are tissue specific and in part due to glucocorticoid secretion
• Changes are in part reversible and represent switches in metabolic strategy

## Teaser

Cai et al. found increases in mtDNA and a reduction in telomeric DNA in cases of major depression using whole-genome sequencing. Both changes are depression state dependent. Mice exposed to chronic stress or glucorticoids showed that these changes reflect switches in metabolic strategy and are tissue specific and partial reversible.

## Introduction

Adverse life experiences, particularly those in childhood, contribute to disease morbidity and mortality [1–7]. There is considerable interest in understanding the mechanisms through which they do so, as it remains unclear how illness becomes apparent decades after the presumed initiating event. Long-standing hypotheses include chronic activation of the hypothalamic-pituitary-adrenal axis [8–10] and alterations of neuroimmune function [11]. Molecular signatures of stressful life experiences and their relation to disease are therefore of special interest to clarify the causal relationship between signature, disease, and stress.

Causal associations between stressful life events and early adversities such as childhood sexual abuse and major depression (MD) are well documented [12–14], suggesting that molecular signatures of stress may be enriched in sufferers of MD. The China, Oxford and VCU Experimental Research on Genetic Epidemiology (CONVERGE) recruited 5,864 women with recurrent MD and 5,783 matched controls, from whom low-coverage genome sequences were obtained together with aggregate measures of lifetime adversities, including assessments of childhood sexual abuse [15, 16] and stressful life events [17, 18]. In CONVERGE, both childhood sexual abuse and stressful life events are strongly associated with risk for MD. More severe forms of abuse are more strongly associated with MD than milder forms, consistent with a causal relationship [16, 18–20].

We focused on two variable components of the somatic genome suspected to be associated with adverse life experiences: telomeric DNA and mtDNA. Accelerated shortening of telomeres, the sequence that caps the ends of chromosomes, has been associated with stress [21–23], anxiety [24], and MD [25] (although not all findings have been replicated [26, 27]). Abnormal mitochondrial morphology and altered metabolic activity has been reported in mood disorders [28]. Our aim was to establish whether telomere length and the amount of mtDNA represent markers of stress-related illness and to explore how such molecular signatures might arise.

## Results

### Shortened Telomeres and Increased mtDNA Are Associated with Adversity

**Figure 1:**
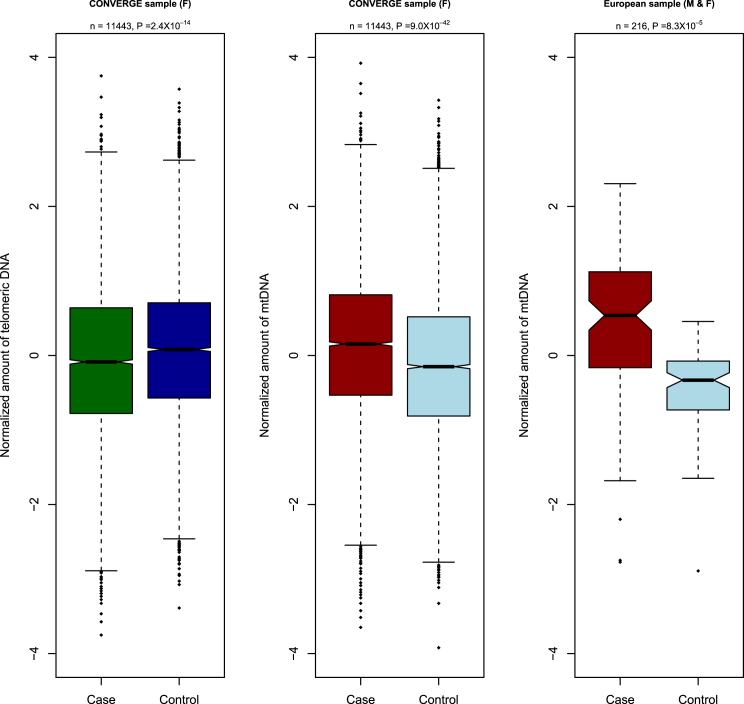
Two Molecular Markers of Depression: Mitochondrial DNA and Telomere Length Left: Boxplot of normalized measure of mean telomere length (vertical axis) for cases and controls in the CONVERGE study. Middle: Boxplot of the normalized amount of mtDNA (vertical axis) in cases and controls in the CONVERGE study. Right: Boxplot of the normalized amount of mtDNA (vertical axis) in cases and controls in the GENDEP/DECC studies (labeled IOP).

We first examined the relationship between mean telomere length and amount of mtDNA with MD. We assessed the mean length of telomeres (across all chromosomes) from low-coverage whole-genome sequencing (mean coverage of 1.7X) of saliva DNA samples of 11,670 subjects (Experimental Procedures). MD was associated with shorter mean telomere length: in a logistic regression model, the odds ratio for the contribution of normalized measure of mean telomere to the risk of MD is 0.85 (95% CI = 0.81–0.89, p = 2.84 × 10^−14^). Figure 1A shows the normalized distributions of mean telomere sequence length in cases and controls.

We obtained a mean coverage of 102X for the mitochondrial genome from which we estimated for each individual the amount of mtDNA. We observed a highly significant association between MD and the amount of mtDNA (p = 9.00 × 10^−42^ from logistic regression). Cases had more mtDNA than controls: the odds ratio for the contribution of normalized amount of mtDNA to the risk of MD was 1.33 (95% CI = 1.29–1.37). Note that the effect is in the opposite direction to that observed for telomeric DNA. Figure 1B shows the distributions of normalized amount of mtDNA coverage for the cases and controls.

We replicated the association between MD and increased amounts of mtDNA in a European case-control study [29, 30]. In contrast to the CONVERGE sample, the DNA was extracted from blood, and samples were of both sexes. We obtained quantitative PCR (qPCR) measures of mtDNA from 216 individuals (108 cases and 108 controls, 123 women and 93 men). In a logistic model, the odds ratio for the normalized measure of mtDNA’s contribution to the risk of MD was 1.35 (95% CI = 1.11–2.10, p = 8.3 × 10^−5^; Figure 1C).

We next explored the association in the CONVERGE data between stressful life events and both mean telomere length and amount of mtDNA. Telomere length was significantly shorter in those who had experienced more stressful life events (p = 0.0018, by linear regression) and in those reporting childhood sexual abuse (p = 0.043, by linear regression) (Table 1). The amount of mtDNA was significantly correlated with both the total number of stressful life events (linear regression p = 4.83 × 10^−4^) and childhood sexual abuse (linear regression p = 3.65 × 10^−5^). The association of both molecular markers with childhood sexual abuse was stronger with increasingly severe abuse (Table 1).

### Molecular Changes Are Not Due to Technical or Biological Artifacts

We explored a number of explanations for the association between molecular markers and MD (Figures 1, S1, and S2; Tables 1 and S1; Supplemental Experimental Procedures). First, we considered artifacts arising from incorrectly mapped reads. We found that the association between amount of mtDNA and MD could not be explained by contamination or mapping errors: none of the reads used for assessing the amount of mtDNA mapped to a set of all bacterial and plasmid genomes, and none mapped to nuclear copies of mtDNA.

Second, we considered whether the molecular changes might be due to medication. We could not explain the telomere length or mtDNA changes as a result of cases taking antidepressant medication: among the MD cases, 975 reported never having taken any antidepressants. Neither the amount of mtDNA nor telomeric length in these subjects differed significantly from that assayed in the 4,861 individuals reporting taking antidepressants (t test p = 0.96 and p = 0.88, respectively).

Third, we considered whether the effects might be explained by alterations in the cellular composition of the saliva between cases and controls (see Supplemental Experimental Procedures). Methylation of cytosine residues at cytosine-guanine (CpG) dinucleotides differs between cell types [31–35] and thus contains information about the cellular composition of the tissue from which it was extracted [36–38]. We assessed methylation in 156 individuals (78 cases and 78 controls), selected from the extremes of the distribution of amount of mtDNA, and matched for age and other potential confounds. The sites assayed are shown in Table S1, and the percentage of methylation at each CpG site is shown in Figure S1. MD case-control status remained highly significantly associated with the amount of mtDNA (t test p value = 5.14 × 10^−18^) and telomere length (p = 6.83 × 10^−5^) after accounting for the degree of methylation at each of the sites (Figure S2). Expressed as a change in effect size using Nagelkerke’s R^2^ measure, there is a 6% reduction in the R^2^ in a model including methylation and the amount of mtDNA to predict MD and a 9% reduction for telomere length. From this analysis, we concluded that the cellular composition of saliva collected from cases differed slightly from that of controls and explained less than 10% of the differences in the amount of mtDNA and telomere length between cases and controls.

### Molecular Changes Are Contingent on the Depressed State

To investigate a causal relationship between stressful life events, MD, amount of mtDNA, and telomere length, we performed a series of conditional regression analyses, assuming that stressful life events preceded the onset of MD and the molecular changes (Supplemental Experimental Procedures). Table 2 shows the counts of individuals categorized by MD disease status and number of stressful life events, with the means and SEs for the amount of mtDNA (Table 2) and telomere length (Table 2) within each category.

If stressful life events have independent causal effects on MD and the molecular measures, then the latter should become independent of MD after conditioning on the number of stressful events. Table 2 shows this is not the case because the mean differences in amount of mtDNA and telomere length between cases and controls, when stratified for the number of stressful life events, remained highly significant (t tests in third column of Table 2; mtDNA p values range from 1.25 × 10^−18^ to 0.37; telomere length p values range from 4.23 × 10^−5^ to 0.0083).

We next asked whether the effect of stressful life events on MD is entirely indirect, acting via changes in the amount of mtDNA or telomere length. We rejected this explanation because the association between MD and stressful life events remains highly significant after conditioning on either amount of mtDNA (p = 5.60 × 10^−99^; see Table S2, i) or telomere length (p = 2.x10^−100^; Table S3, i) in a logistic regression model. In contrast, the association between stress and amount of mtDNA or telomere length disappeared when conditioned on MD (p = 0.11 Table S4, i, and p = 0.11 Table S5, i, respectively). In other words, the predictive power of stress on amount of mtDNA and telomere length is mediated through a history of MD.

These conclusions also hold when the number of stressful life events is replaced by a history of childhood sexual abuse. In particular, there was no significant difference in the amount of mtDNA or telomere length when comparing controls who reported a history of childhood sexual abuse with those who did not. Mean values of normalized mtDNA for controls who reported any form of childhood sexual abuse was −0.136 (SE = −0.007) and −0.095 (SE = −0.001) for no such history; t test p value = 0.66. Comparable values for telomere length were 0.168 (SE = 0.0125) and 0.072 (SE = 0.001); t test p value = 0.27.

These analyses indicate that the molecular markers represent the current state of illness, regardless of the path by which it is reached, and predict that the most pronounced changes would be found in subjects currently reporting a severe mood disorder. Our analyses up to this point used subjects for whom we did not have a current state measure of mood. We therefore measured the amount of mtDNA in a separate Chinese case-control cohort of MD [39] where a state measure of mood was available (the Hamilton rating scale [40]). We selected 29 cases with scores greater than 25 (very severe) and 25 controls with scores of less than 5. Despite using such a small sample, we observed a highly significant difference (t test p value = 0.0008) and an odds ratio of 2.94 (95% CI 1.26–6.02), more than twice the odds ratio seen in the CONVERGE sample (odds ratio = 1.33).

### Stress Increases the Amount of mtDNA and Shortens Telomeres

To gain a mechanistic understanding of the relationship between stress, amount of mtDNA, and telomere length, we undertook a mouse experiment. Sixteen C57BL/6J mice (eight males and eight females) were stressed for 4 weeks (for 5 days, a different stressor was administered: tail suspension, force-swim, foot shock, restraint, and sleep deprivation, followed by 2 days rest). After 0, 2, and 4 weeks of stress, amount of mtDNA and telomere length were assessed by qPCR and compared to age-matched non-stressed controls (eight males and eight females).

**Figure 2:**
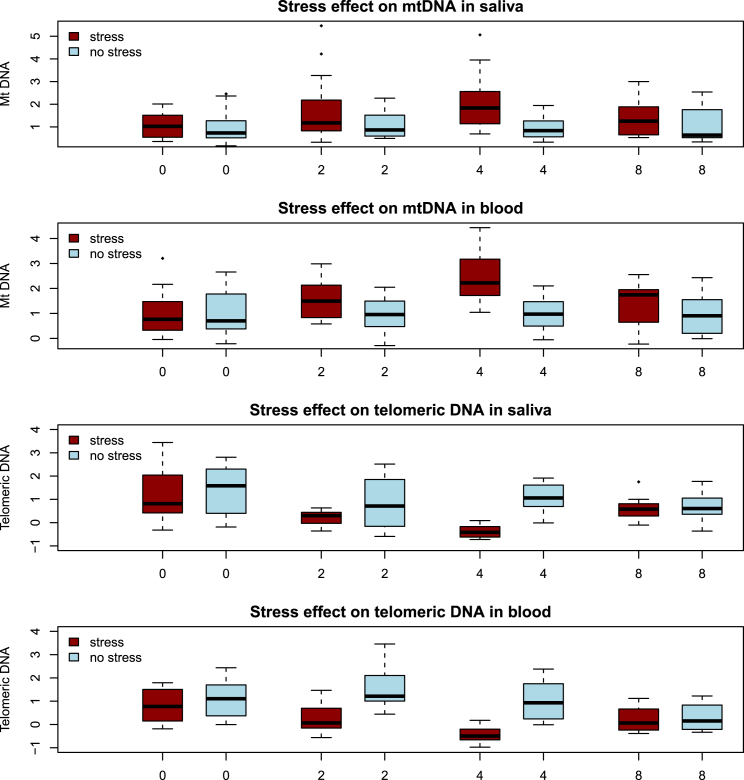
Effect of Chronic Stress on mtDNA in Saliva and Blood of Mice Boxplot of relative mtDNA changes and relative mean telomere length over time in mice exposed to stress (red) and controls (blue). The vertical axis shows the amount of DNA, assessed by qPCR, relative to the mean of the values obtained before stress was imposed (week 0). The mean of week 0 is set to 1, so that results from subsequent weeks are fold changes relative to pre-stress levels. The horizontal axis is time in weeks from the beginning of the experiment. Stress was discontinued after week 4, so week 8 shows results for previously stressed animals after 4 weeks of living in a home cage. At the 4 week time point, the amount of mtDNA in blood and saliva was significantly greater in stressed animals (t test p = 6.1 × 10^−5^ and p = 0.0036, respectively). Also at the 4 week time point, relative mean telomere lengths in stressed mice were significantly lower in saliva (t test p = 0.0001) and blood (t test p = 0.0017) as compared to non-stressed mice. Differences between stressed and non-stressed mice in both measures were not significant at the start of the experiment or at the 8 week time point.

Consistent with our findings in humans, in mice, stress significantly increased the amount of mtDNA and decreased telomere length in saliva and in blood (Figure 2). After 4 weeks of stress, there was a mean increase in the amount of mtDNA of 210% compared to the unstressed animals in saliva (t test p = 0.0036) and 240% in blood (t test p = 6.1 × 10^−5^). At the same time, the length of telomeric DNA was reduced 28% in saliva (t test p = 0.0001) and 30% in blood (t test p = 0.0017) in stressed mice as compared to non-stressed. There were no significant differences in the white cell parameters between stressed and non-stressed animals (all p values > 0.05), indicating that this result is unlikely to be due to differences in the blood cellular composition.

After 4 weeks of stress, half of the animals (eight stressed mice and eight controls) were kept in home cages without any intervention to model a recovery period of no stress. Molecular markers were again tested in blood and saliva, and results are shown as week 8 in Figure 2. Four weeks after the discontinuation of stress, there were no significant differences between control animals and those that had been previously exposed to stress (amount of mtDNA in saliva p = 0.50, in blood p = 0.38; telomere length in saliva p = 0.85, in blood p = 0.76; all p values from t tests). These results indicate that the molecular changes are, at least in part, reversible.

**Figure 3:**
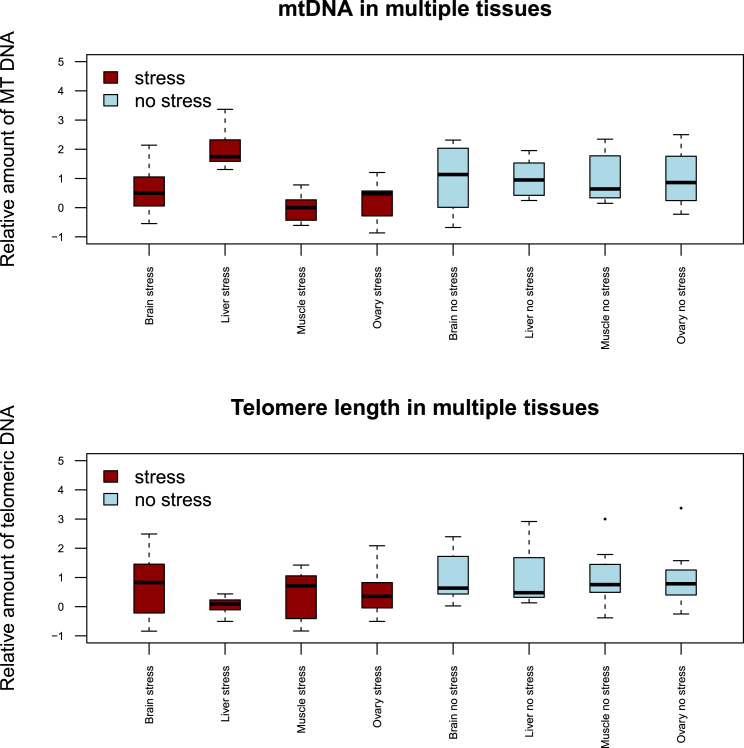
Alterations of mtDNA in Different Tissues after 4 Weeks of Stress Top: Assessment of mtDNA in four tissues. Bottom: Assessment of telomere length in four tissues. The vertical axis shows the amount of mtDNA or telomere length assessed by qPCR, relative to the mean of the values obtained for control animals (no stress). The horizontal axis gives the names of the tissues for the two conditions: stress (red) and no stress (blue).

Immediately after the cessation of stress, multiple tissues from the other 16 animals were assayed for the amount of mtDNA and telomere length. Figure 3 shows results for four tissues: liver, muscle, brain (hippocampus), and ovary (ovary was chosen as we were interested to assess whether the changes might be transmitted to the next generation). For the amount of mtDNA, there was a significant increase in liver (p value from t test = 0.005), a significant decrease in muscle (p = 0.014), but no significant alterations in hippocampus (p = 0.50), and a suggestive change in ovary (p = 0.086). For telomere length, there was a significant 54% reduction in liver (p = 0.03), but no significant changes in other tissues (muscle p = 0.23, hippocampus p = 0.59, ovary p = 0.30). These results reveal tissue-specific changes in mtDNA, and possibly in telomere length, as a consequence of stress.

### Mitochondrial Function Is Altered in Tissues with Increased mtDNA

**Figure 4:**
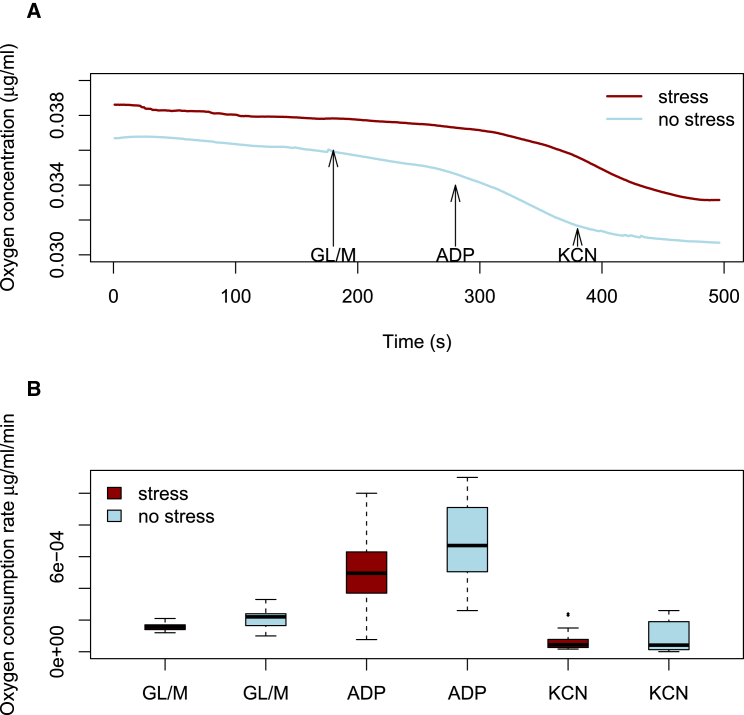
The Oxygen Consumption of Mouse Liver after Stress Administration (A) Oxygen concentration (vertical axis) detected per second (horizontal axis) per μg of mitochondria. The slope of the curve indicates the rate of oxygen consumption. Glutamate/malate (GL/MA) is added at 3 min after addition of isolated mitochondria, and oxygen consumption was assessed after substrate addition. The addition of ADP (100 s later) initiates active respiration while potassium cyanide (KCN) (100 s later) inhibits all mitochondrial function. (B) Oxygen consumption rate per μg of mitochondria after the addition of the three compounds, comparing stressed and non-stressed animals.

Altering the amount of mtDNA presumably reflects functional changes in mitochondria, a hypothesis we tested by measuring and comparing the oxidative phosphorylation (OXPHOS) capacity of mitochondria-enriched fractions from the liver of stressed and non-stressed mice. Figure 4A shows the mean values for the change in oxygen concentration over time for liver mitochondrial preparations taken from eight stressed and eight control (not stressed) animals. Addition of an equal amount of ADP (driving force for the electron transport chain after depletion of residual driving force in the fraction with excess glutatmate and malate) induced a greater increase in oxygen consumption in mitochondria from the non-stressed animals than stressed ones (p = 0.038, from a linear model; Figure 4B). The complete quenching of OXPHOS upon addition of electron transport chain inhibitor potassium cyanide in both stressed and non-stressed mice showed oxygen consumption during the experiment was solely due to OXPHOS. Results of this experiment showed that OXPHOS efficiency was reduced in the liver tissue of mice whose amount of mtDNA had increased in response to stress, suggesting either an adaptive switch to glycolysis or a compromise in mitochondrial function.

### Glucocorticoid Administration Reproduces the Effects of Stress

**Figure 5:**
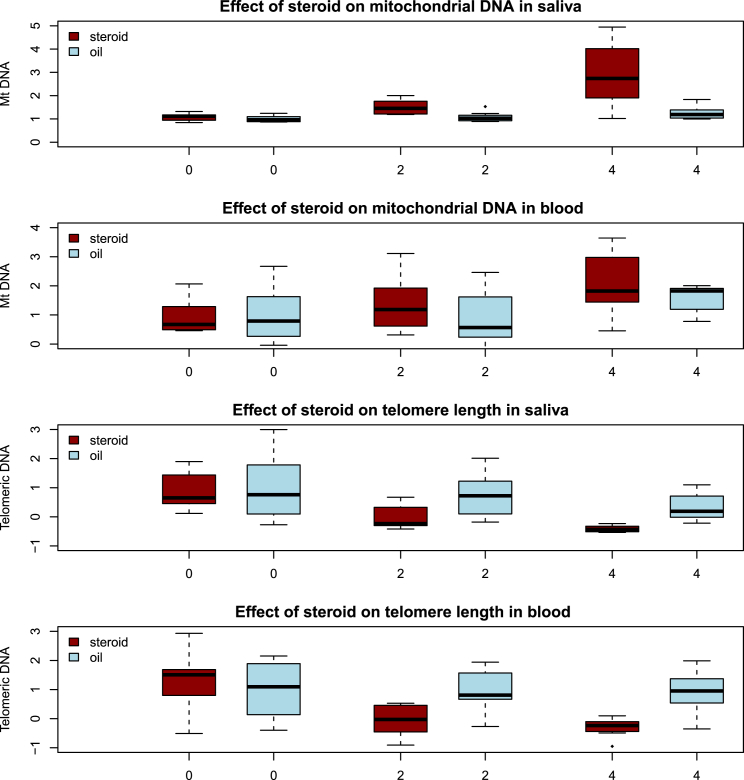
Effect of Daily Subcutaneous Injection of Corticosterone on mtDNA and Telomere Length in Saliva and Blood in Mice Boxplot of relative amount of mtDNA and relative mean telomere length over time in mice injected with corticosterone (red) and controls injected with the same volumes of oil vehicle (blue). The vertical axis shows the amount of mtDNA assessed by qPCR, relative to the mean of the values obtained before corticosterone was injected (week 0). The mean of week 0 is set to 1, so that results from subsequent weeks are fold changes relative to pre-stress levels. The horizontal axis is time in weeks from the beginning of the experiment. After 4 weeks, the amount of mtDNA levels in mice injected with corticosterone was significantly higher in saliva (t test p = 0.011) and blood (t test p = 0.0013) as compared to mice injected with oil vehicle; relative mean telomere lengths were significantly reduced in both tissues (in saliva: t test p = 0.0023; in blood: t test p = 0.0016) in mice injected with corticosterone as compared to mice injected with oil vehicle.

What might be inducing the molecular changes? We considered one mechanism: activation of the hypothalamic pituitary adrenal (HPA) axis [41–48]. We administered corticosterone to eight C57BL/6J female mice over 4 weeks and oil vehicles of the same volume to eight control mice of the same strain. Figures 5 shows that after 4 weeks, there was significantly more mtDNA in the saliva (p = 0.011) and in the blood (p = 0.0013) of treated mice compared to controls and that telomere length had significantly reduced in both tissues (in saliva: p = 0.0023; in blood: p = 0.0016; all p values from t tests).

## Discussion

We report here two important observations on the relationship between MD and two molecular signatures of adversity, the amount of mtDNA and mean telomere length. First, the changes in amount of mtDNA and telomere length are contingent on the presence of MD. We found no significant molecular changes in those who reported stressful life events, including childhood sexual abuse, but had never been depressed. Second, in a mouse model, while stress over a period of weeks did increase the amount of mtDNA and shorten telomere length, both changes were at least partly reversible. While early environmental adversity may result in permanent changes in physiology and risk of disease [49], our results indicate that it is important to recognize two trajectories, one leading to molecular signatures of stress and one to illness.

For the first trajectory leading from adversity to molecular changes, one possible pathway is through the endocrine system, particularly the activation of the hypothalamic pituitary axis, since changes in both molecular markers could be reproduced in mice by administration of corticosterone. Release of glucocorticoids is known to increase in response to stress. Severe stressors, such as childhood sexual abuse [42, 50], alter pituitary-adrenal and autonomic reactivity. In some circumstances, the consequences may be deleterious rather than adaptive: glucocorticoids have been implicated in the pathophysiology of posttraumatic stress disorder [51, 52], and it has been known for many years that some patients with MD exhibit hypersecretion of cortisol [41, 53, 54], in part due to corticotrophin releasing factor (CRF) hypersecretion [55].

For the second trajectory leading to illness, we hypothesize that while adversity may on its own have an effect on both the amount of mtDNA and mean telomere length, the extent and persistence of these molecular changes depend on an individual’s susceptibility to MD, either from genetic or additional environmental predisposing factors. In many individuals, the molecular signatures will be small and transitory, but in those with MD, the effects may be larger or last for a longer period of time. Subjects who have never been diagnosed with MD, yet suffered severe adversity, may have had detectable alterations in mtDNA levels and mean telomere length in particular tissues at the time they experienced stressful life events, but these changes would have reversed and no longer be detectable by the time they were interviewed. We emphasize that the molecular changes we observe are neither risk factors nor causes of MD. The correlation between stress, mtDNA, and telomere length is contingent upon MD; we could find no evidence that stressful life events act via changes in mtDNA or telomere length to increase the risk of MD. Thus, our data provide no support for a role of changes in the amount of mitochondrial DNA or length of telomeres in regulating mood.

The disease-state dependence of the measures is important when considering the potential use of the changes as biomarkers. It is noteworthy in this regard that in a sample when we assayed amount of mtDNA in currently severely ill subjects, a robust difference was detected in a comparison of just 29 cases and 25 controls. This suggests that, despite the relatively small effects and large variances seen in the saliva sample, there may be circumstances where the amount of mtDNA could serve as a useful biomarker. The relatively larger increases seen in the mouse experiment (up to 4-fold) suggest that controlling for inter-individual variation would improve the chances of the biomarkers having a clinical application.

Changes in mean telomere length and levels of mtDNA presumably reflect altered metabolic strategies in times of perceived or expected stress. Experiments assessing OXPHOS efficiency in mice showed a decrease in OXPHOS energy production in stressed mice with elevated mtDNA levels. The tissue-specific effects of stress on amount of mtDNA and mean telomere suggest different, or possibly sequential, pathways governing tissue-specific change. It is possible that these changes might in part explain changes in appetite and sleep occurring during the state of depression.

## Experimental Procedures

### The CONVERGE Study, Samples, DNA Preparation, and Sequencing

All 11,670 samples are drawn from the CONVERGE study of MD. The study protocol was approved centrally by the Ethical Review Board of Oxford University (Oxford Tropical Research Ethics Committee) and the ethics committees responsible for each hospital in China. The study posed minimal risk to the subjects (an interview and saliva sample). Stressful life events and childhood sexual abuse were assessed retrospectively. The stressful life events section of the CONVERGE interview was developed for the Virginia Adult Twin Study of Psychiatric and Use Disorders (VATSPUD) [17]. It assesses 16 traumatic lifetime events and the age at their occurrence. The childhood sexual abuse was a shortened version of the detailed module used in the VATSPSUD study, which was in turn based on the instrument developed by Martin et al. [56]. DNA was extracted from saliva samples using the Oragene protocol.

Sequencing libraries were constructed from DNA fragmented using the Covaris Adaptive Focused Acoustics (AFA) technology. QIAquick Gel Extraction kit was used to purify the DNA fragments. Each DNA sample was uniquely tagged with a sequencing index for multiplex library preparation. Insert sizes were on average 400 bp. Library quality was checked with an Agilent 2100 Bioanalyzer and ABI StepOnePlus Real-Time PCR System. Libraries were sequenced on Illumina Hiseq 2000 machines. Sequencing reads for each of the 11,670 samples were aligned to Genome Reference Consortium Human Build 37 patch release 5 (GRCh37.p5) with Stampy (v.1.0.17) [57] and stored in BAM format [58].

### The GENDEP and DeCC Studies, Samples, and qPCR

Cases and control samples were drawn from the United Kingdom Depression Case-Control (DeCC) study [29] and the Genome-Based Therapeutic Drugs for Depression (GENDEP) study [30]. mtDNA copy number was estimated from DNA extracted from blood samples by qPCR, using a TaqMan Universal PCR MasterMix on an ABI StepOnePlus Real-Time PCR System (Life Technologies). The pre-designed TaqMan assay Hs02596867_s1 was used to amplify a fragment of the MT-CYB gene on the mitochondrial chromosome in duplex with the TaqMan RNaseP Copy Number Reference Assay (Life Technologies, part number 4403326) as an internal control.

### Extracting and Quality Control of Mitochondrial Reads from Low-Coverage Whole-Genome Sequencing Data

All reads mapped to the human mitochondrial genome NC_012920.1 were extracted from the whole-genome BAM files mapped to GRCh37.p5 using Samtools (v.0.1.18) [58]. The mitochondrial reads extracted were then converted to the FASTQ format using Picardtools (v.1.108, http://broadinstitute.github.io/picard/) and mapped to a combined reference containing 894 complete bacterial genomes, 2,024 complete bacterial chromosomes, 154 draft assemblies, and 4,373 complete plasmids sequences (in total, 7,390 unique bacterial DNA sequences) available on NCBI using BWA (v.0.5.6) [58]. All reads mapped to bacterial DNA sequences were filtered out using Samtools (v.0.1.18) [58] by imposing a mapping quality filter of 59 (Phred-scale probability of being wrongly mapped) and removing reads with FLAGs (-F 1804) that identify unmapped reads, unpaired reads, reads that do not pass quality control, reads that may be PCR or optical duplicates, and reads that are secondary alignments that also map to other areas of the reference. No reads from filtered BAM files of any sample map onto the combined bacterial reference.

### Estimation of mtDNA Copy Number

Average read depth per 100 bp is calculated for the mtDNA reads mapped to NC_012920.1 both before and after filtering out poorly mapped reads including those potentially from bacterial genomes using SAMTOOLs (v.0.1.18) [58]. There are regions in the mitochondrial genome replicated in the nuclear genome commonly known as nuclear copies of mitochondrial DNA (NUMTs), which would most likely be present as secondary alignments. We calculated average read depth per segment of 100 bp in the mtDNA alignments both before and after filtering and compared the two sets of read depths. To reduce errors in estimation of coverage due to NUMTs, segments with big differences in read depth (>5% of the filtered read depth) between the filtered and unfiltered alignments that are more likely to span NUMTs were excluded from our calculation of mtDNA copy number. We arrived at a measure of mtDNA copy number by taking the mean read depth in the filtered alignments across all remaining 100 bp segments, then regressing it with sequencing batch, sample age, and average filtered read depth on chromosome 20, then transforming the residuals to normality using a quantile normal function in the R statistical software language [59].

### Estimation of Mean Telomere Length

Mean telomere length was quantified from low-coverage whole-genome sequencing data mapped to Genome Reference Consortium Human Build 37 patch release 5 (GRCh37.p5) with Telseq v.0.0.1 [60]. The estimated mean telomere length output from Telseq was already corrected for whole-genome coverage and the GC content of DNA; it was then regressed with batch and sample age before the residuals were transformed to normality using a quantile normal function in the R statistical software language [59].

### Association between Molecular Markers, MD, and Stress

We tested for association between MD and molecular markers using logistic regression in the R statistical software language [59]. All logistic regression models included as covariates the first three principal components (PCs) from a principal-component analysis (PCA) performed with Genome-wide Complex Trait Analysis (GCTA) v.1.24.4 [61] using a genetic relationship matrix (GRM). The GRM was generated with 561,819 common, tagging single nucleotide polymorphisms (SNPs) from all autosomes. All SNPs in this tagging set were polymorphic in 1,000G phase 1 Asian (ASN) panel, occur at greater than 5% minor allele frequency in CONVERGE study samples, and are out of linkage disequilibrium (LD) with each other (maximum pairwise LD = 0.8).

### Mouse DNA Extraction

DNA was extracted from mouse tissues using a QIAamp DNA Investigator Kit (QIAGEN). Saliva was collected from mice by inserting a disposable inoculation loop into the animal’s mouth, allowing the animal to chew for a few seconds, before rinsing the loop in QIAamp DNA Investigator Kit ATL buffer. Blood was collected from a superficial tail vein.

### Quantification of mtDNA Levels by qPCR

qPCR was carried out using the Bio-Rad iQ SYBR Green super mix supplied by Roche Molecular Biochemicals. A nuclear genomic fragment of 160 bp was amplified from the mouse *Gapdh* gene (forward 5′-TGACGTGCCGCCTGGAGAAAC-3′, reverse 5′-CCGGCATCGAAGGTGGAAGAG-3′. A 117 bp fragment of the mitochondrial genome (positions 13603–13719) was amplified with primers described in [62] (forward 5′- CCCAGCTACTACCATCATTCAAGT-3′, reverse 5′-GATGGTTTGGGAGATTGGTTGATGT-3′). qPCR was performed under the following conditions: denaturation 95°C for 10 min followed by 50 cycles of 15 s at 95°C and 1 min at 60°C. An estimate of the mtDNA copy number was calculated using the mean of Gapdh as a control [63]. All samples were duplicated at each time point. PCR efficiencies were between 90%–110% (average coefficient variance: 0.806). PCR runs were discarded if they failed to meet the following criteria: no template control (NTC) with a quantitation cycle (Cq) < 38 cycles; sample with a Cq > 30 cycles; PCR efficiency > 90% and < 110.0%; standard curve R^2^ < 0.980; replicate group Cq SD greater than 0.20. qPCRs were carried out at the end of each experiment and all time points were analyzed on a single plate, thus excluding batch effects.

### Quantification of Telomere Length by Monochrome Multiplex qPCR

Average telomere length was measured from mouse DNA using a previously described monochrome multiplex qPCR (MMQPCR) method [64] with the following conditions: denaturation at 95°C for 15 min followed by 2 cycles of 15 s at 94°C and 60 s at 49°C, 4 cycles of 15 s at 94°C and 30 s at 59°C, 20 cycles of 15 s at 85°C and 30 s at 59°C, and 27 cycles of 15 s at 94°C, 10 s at 84°C, and 15 s at 85°C. Forward and reverse telomeric primers were 5′-ATACCAAGGTTTGGGTTTGGGTTTGGGTTTGGGTTCATGG-3′ and 5′-GAGGCAATATCCCTATCCCTATCCCTATCCCTATCCCTAACC-3′. Average telomere length ratio was estimated from the ratio of telomere product to that of a single copy nuclear gene albumin, forward and reverse primers for which were 5′-CGGCGGCGGGCGGCGCGGGCTGGGCGGAAACGCTGCGCAGAATCCTTG-3′ and 5′-GCCCGGCCCGCCGCGCCCGTCCCGCCGCTGAAAAGTACGGTCGCCTG-3′.

### Mitochondrial Oxygen Consumption

We measured oxygen consumption from mouse liver mitochondrial preparations over time using a Clark electrode. After the addition of respiratory substrates (glutamate and malate), oxygen consumption was monitored for 100 s, after which ADP was added and oxygen consumption measured for a further 100 s. Potassium cyanide (KCN) was added 100 s later to inhibit all mitochondrial oxygen consumption.

### Animal Experiments

All experiments were carried out in strict accordance with the recommendations in the Guide for Laboratory Animals Facilities and Care as promulgated by the Council of Agriculture, Executive Yuan, ROC, Taiwan. The protocol was approved by the Institutional Animal Care and Use Committee of Chang Gung University (permit number: CGU13-067). Animals were group housed and randomly assigned to stress or non-stress experiments.

Mouse stress experiment: mice (strain C57BL/6J, female n = 8, male n = 8, aged 12 months) were stressed over 5 days followed by 2 days rest, repeated for 4 weeks. On the first day, animals were suspended from their tails for 10 min. This was repeated three times, with 5 min rest between tail suspensions. On the second day, animals were placed in a cylinder of deep water from which there was no escape for 10 min. The forced swim was repeated twice with a 10 min rest. On the third day, a foot shock was administered three times (0.75 mA for 10 s with 10 s rest). On the fourth day, animals were restrained in a cylindrical tube (12 cm in length and 3 cm in diameter) for 3 hr. On the fifth day, animals were sleep deprived for 24 hr (mice were put in water tank, containing multiple and visible platforms [4.5 cm in height and diameter] surrounded by water for 24 hr). For the glucocorticoid experiment, mice (strain C57BL/6J, female n = 8, aged 12 months) underwent daily subcutaneous injection of 30 mg/kg corticosterone (Sigma) or vehicle (oil) for 28 days. Association between mtDNA, telomere length, and stress was performed in a linear mixed model using the lme4 package in the statistical software language R [59]. The null model included only weight. Variation in the amount of mtDNA between different tissues was assessed by a t test, comparing values between controls and experimental animals.

## Author Contributions

N.C., Yihan Li, S.C., K.K., R.M., and J.F. prepared the manuscript. Y.C., Yihan Li, H.D., B.D., Keqing Li, W.S., J.G., B.H., S.G., Jian Hu, C.H., G.H., G.J., Youhui Li, Kan Li, Yi Li, G.L., L. Liu, T.L., Ying Liu, L. Lv, H.M., H.S., J. Shen, J. Shi, J. Sun, M.T., Xumei Wang, Gang Wang, Xueyi Wang, J.Y., K.Z., N.S., J.Z., Z.Z., W.Z., H.Z., J.F., and K.K. handled sample collection. S.C., J.F., J.N., H.-Y.H., Y.-T.L., and G.-J.H. carried out animal experiments. N.C., Yihan Li, J.N., G.B., M.R., K.K., R.M., and J.F. handled human mtDNA and telomere analyses. Q.L., Jingchu Hu, W.K., W.J., Yihan Li, Guangbiao Wang, L.W., P.Q., Yuan Liu, T.J., Y. Lu, X.Z., Y.Y., Yingrui Li, H.Y., Jian Wang, X.G., R.M., J.M., J.F., Jun Wang, and X.X. carried out genome sequencing and analysis. N.C., Yihan Li, J.F., and R.M. carried out genetic analysis. N.C. identified the shortened telomeres and examined the relationship between both molecular markers with stress and depression. S.C. performed all animal experiments and analyzed the qPCR and OXPHOS output data. Yihan Li identified the excess of mtDNA in low-coverage sequencing data in cases of MD as compared to controls.

## Figures and Tables

**Table 1:** Relationship between Childhood Sexual Abuse, Telomere Length, and the Amount of Mitochondrial DNA

CSA Type	Excess Telomeric DNA^a^	t Value^b^	p Value^c^	Excess mtDNA^a^	t Value^b^	p Value^c^	Number Cases^d^	Number Controls^e^	Total^f^
Non-genital CSA	0.02	0.35	0.73	0.08	1.37	0.169	186	81	267
Genital CSA	−0.08	−1.27	0.20	0.11	2.02	0.045	240	47	287
Intercourse CSA	−0.20	−2.45	0.01	0.38	4.67	3.05 × 10^−6^	159	17	176

Results for analysis of variance in which different forms of childhood sexual abuse (CSA) predict telomere length and the amount of mtDNA. Non-genital CSA refers to sexual invitation, sexual kissing, and exposing; genital CSA refers to fondling and sexual touching; and intercourse CSA refers to attempted or completed intercourse.

a Estimated excess of telomeric or mtDNA over mean telomeric DNA or mtDNA in individuals with no CSA.

b t statistic of tests of hypotheses that underlying excess is zero.

c p value of tests of hypotheses that underlying excess is zero.

d Number of MD cases.

e Number of controls.

f Number of total individuals.

**Table 2:** Relationship between Stressful Life Events, mtDNA, Telomere Length, and Major Depression

Difference in Normalized mtDNA Levels in Cases of MD and Controls per #SLE			
#SLE	MD Control	MD Case	mtDNA Difference t Statistic, p Value
0	−0.132 (0.019), 2,487	0.142 (0.024), 1,689	−8.86, 1.25 × 10^−18^
1	−0.156 (0.026), 1,432	0.0987 (0.027), 1,441	−6.83, 1.01 × 10^−11^
2	−0.103 (0.034), 757	0.165 (0.033), 935	−5.65, 1.84 × 10^−08^
3	−0.068 (0.058), 334	0.085 (0.044), 507	−2.12, 0.03
4+	0.062 (0.067), 221	0.132 (0.040), 666	−0.89, 0.37

**Difference in Telomere Length in Cases of MD and Controls per #SLE**			

**#SLE**	**MD Control**	**MD Case**	**Telomere Difference T Statistic, p Value**

0	0.078 (0.020), 2,542	−0.053 (0.025), 1,722	4.10, 4.23 × 10^−5^
1	0.098 (0.026), 1,461	−0.048 (0.027), 1,470	3.91, 9.42 × 10^−5^
2	0.093(0.035), 780	−0.069 (0.032), 952	3.36, 8.05 × 10^−4^
3	0.042 (0.053), 342	−0.085(0.045), 517	1.82, 0.069
4+	0.060 (0.060), 229	−0.129 (0.038), 677	2.65, 0.0083

For each category of stressful life event (#SLE, ranging from none [0] to more than four [4+] reported events), Table 2 reports the means and SEs of the normalized mtDNA levels (top section of the table) and normalized telomere length measures (bottom section of the table), followed by the numbers of individuals, for MD cases and controls. The last column gives the t statistic and p value for the difference between cases and controls.

## Supplemental Information

Document S1. Supplemental Experimental Procedures, Figures S1 and S2, and Tables S1–S5

Document S2. Article plus Supplemental Information
