# Supplementary material for: Molecular Signatures of Major Depression
Source: Curr Biol. 2015 May 4;25(9):1146–56. doi: 10.1016/j.cub.2015.03.008 (PMC4425463; doi:10.1016/j.cub.2015.03.008)
Supplement: Document S1. Supplemental Experimental Procedures, Figures S1 and S2, and Tables S1–S5 [file mmc1.pdf]

Current Biology

Supplemental Information

Molecular Signatures of Major Depression

Na Cai, Simon Chang, Yihan Li, Qibin Li, Jingchu Hu, Jieqin Liang, Li Song, Warren Kretzschmar, Xiangchao Gan, Jerome Nicod, Margarita Rivera, Hong Deng, Bo Du, Keqing Li, Wenhu Sang, Jingfang Gao, Shugui Gao, Baowei Ha, Hung-Yao Ho, Chunmei Hu, Jian Hu, Zhenfei Hu, Guoping Huang, Guoqing Jiang, Tao Jiang, Wei Jin, Gongying Li, Kan Li, Yi Li, Yingrui Li, Youhui Li, Yu-Ting Lin, Lanfen Liu, Tiebang Liu, Ying Liu, Yuan Liu, Yao Lu, Luxian Lv, Huaqing Meng, Puyi Qian, Hong Sang, Jianhua Shen, Jianguo Shi, Jing Sun, Ming Tao, Gang Wang, Guangbiao Wang, Jian Wang, Linmao Wang, Xueyi Wang, Xumei Wang, Huanming Yang, Lijun Yang, Ye Yin, Jinbei Zhang, Kerang Zhang, Ning Sun, Wei Zhang, Xiuqing Zhang, Zhen Zhang, Hui Zhong, Gerome Breen, Jun Wang, Jonathan Marchini, Yiping Chen, Qi Xu, Xun Xu, Richard Mott, Guo-Jen Huang, Kenneth Kendler, and Jonathan Flint

Figure S1

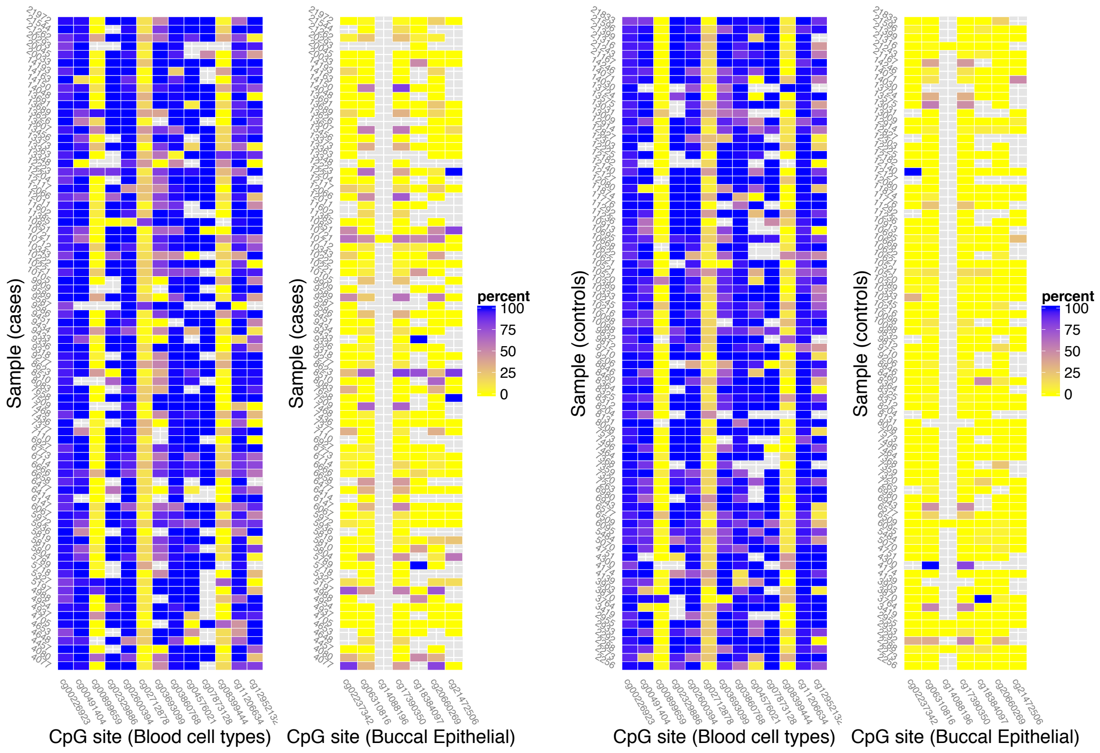

Figure S1 related to Figure 1 and Table 1: Percentage methylation at CpG sites distinguishing between blood cell types and between buccal epithelial and blood cells in 156 samples. Color indicates the percentage methylation where 100% methylated is blue and 0% methylated is yellow. The horizontal axis lists the sites and the vertical axis the identifiers of the samples. Cases are shown on the left and controls on the right.

Figure S2

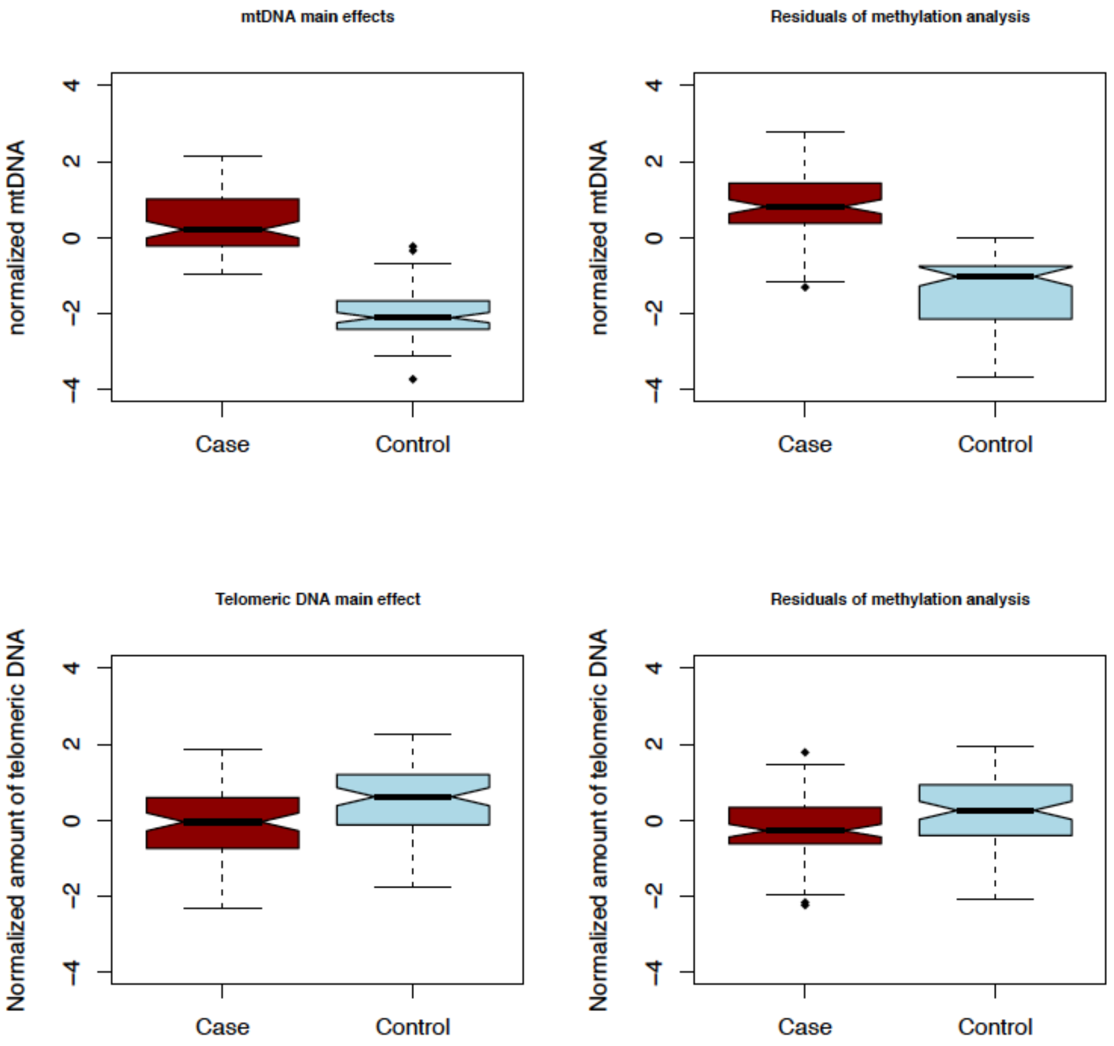

Figure S2 related to Figure 1 and Table 1. Effect of methylation on the relationship between mtDNA and telomeric DNA and case status. Boxplots are shown for mtDNA and telomeric in cases (red) and controls (blue) on the left ("Main effects") and after residuals of both measures are taken from regression on the 20 methylation sites ("Residuals of methylation analysis")

Supplemental Tables

Table S1 related to Figure 1 and Table 1: Buccal epithelial and blood cell types distinguished by different methylation states at 20 CpG sites

CpG site	Gene	Unmethylated cell type	Methylated cell type
cg02237342	ADORA2A	Blood	Buccal Epithelial
cg20660269	ADORA2A	Blood	Buccal Epithelial
cg06310816	GLI3	Blood	Buccal Epithelial
cg17390350	GLI3	Blood	Buccal Epithelial
cg21472506	OTX1	Blood	Buccal Epithelial
cg14088196	PPFIA1	Blood	Buccal Epithelial
cg18384097	PTPN7	Blood	Buccal Epithelial
cg00226923	FGD2	B cells	All others blood cells
cg00491404	EPS8L3	NK cells	All others blood cells
cg00899659	ZNF22	Eosinophils	All others blood cells
cg02329886	HDC	Basophils	All others blood cells
cg02600394	TXK	T cells and NK cells	All others blood cells
cg02712878	FAIM	All others blood cells	T cells
cg03693099	CEL	Eosinophils	All others blood cells
cg03860768	BLK	B cells	All others blood cells
cg04576021	HLA-DO	B cells	All others blood cells
cg07873128	OSBPL5	CD8+/CD16+ NK cells	All others blood cells
cg08399444	GSG1	Granulocytes + Neutrophils	All others blood cells
cg11206634	SFT2D3	Monocytes	All others blood cells
cg12952132	NCR1	CD16+ NK cells	All others blood cells

Table S2 related to Table 2: Effect of normalised measure of normalized amount of mtDNA (nMT), number of stressful life events (SLE) and their interaction on MD disease risk. Shown are the analysis of deviance tables produced by the anova function to compare the fits of nested generalised linear models in the R statistical package. [a]: specification of the model using the notation of R. [b]: The difference in degrees of freedom between the current model and that on the line above. [c]: the difference in deviance (twice the log-likelihood) between the current model and that on the line above. [d]: P-value of test comparing the fit of the current model to the line above. NULL: model with just top 3 principal components calculated from a GRM computed using 561,819 common tagging SNPs as covariates. All fitted models included these covariates.

**(i) glm(MD ~ nMT*SLE)[a]**	**Df[b]**	**Deviance[c]**	**P[d]**
NULL	10465		
nMT	1	164.50	$1.18x10^{-37}$
SLE	1	445.91	$5.60x10^{-99}$
nMT *SLE	1	4.96	0.026

**(ii) glm(MD ~ SLE*nMT)**	**Df**	**Deviance**	**P**
NULL	10465		
SLE	1	460.83	$3.16x10^{-102}$
nMT	1	149.58	$2.14x10^{-34}$
SLE* nMT	1	4.96	0.026

**(iii) glm(MD ~ nMT*CSA)**	**Df**	**Deviance**	**P**
NULL	11031		
nMT	1	174.91	$6.28x10^{-40}$
CSA	1	288.44	$1.09x10^{-64}$
nMT * CSA	1	0.03	0.86

**(iv) glm(MD ~ CSA*nMT)**	**Df**	**Deviance**	**P**
NULL	11031		
CSA	1	301.39	$1.64x10^{-67}$
nMT	1	161.95	$4.24x10^{-37}$
CSA * nMT	1	0.03	0.86

Table S3 related to Table 2: Effect of normalised measures of mean telomere length (nTel), number of stressful life events (SLE) and their interaction on MD disease risk. See **Table S2** for description.

**(i) glm(MD ~ nTel*SLE)**	**Df**	**Deviance**	**P**
NULL	10688		
nTel	1	62.23	$3.06x10^{-15}$
SLE	1	451.97	$2.x10^{-100}$
nTel *SLE	1	0.71	0.40
**(ii) glm(MD ~ SLE* nTel)**	**Df**	**Deviance**	**P**
NULL	10688		
SLE	1	459.67	$5.66x10^{-102}$
nTel	1	54.53	$1.53x10^{-13}$
SLE* nTel	1	0.71	0.40
**(iii) glm(MD ~ nTel *CSA)**	**Df**	**Deviance**	**P**
NULL	11276		
nTel	1	67.78	$1.83x10^{-16}$
CSA	1	303.47	$5.77x10^{-68}$
nTel * CSA	1	2.36	0.12
**(iv) glm(MD ~ CSA* nTel)**	**Df**	**Deviance**	**P**
NULL	11276		
CSA	1	307.26	$8.65x10^{-69}$
nTel	1	64.00	$1.25x10^{-15}$
CSA * nTel	1	2.36	0.12

Table S4 related to Table 2: Effect of MD, number of stressful life events (SLE) and their interaction on normalised measure of amount of mtDNA (nMT). Shown are Analysis of Variance (ANOVA) tables, generated by the anova() function applied to comparisons of linear models fitted by the R statistical package. [a] : Specification of the linear model fitted in the R language. [b]: Difference in degrees of freedom between current model and modle above (the null model, not shown, fitted just top 3 principal components calculated from a GRM computed using 561,819 common tagging SNPs as covariates). [c]: Additional Sum of squares (variance explained) by the current model compared the line above. [d] : Mean Square (Sum Sq divided by Df). [e]: Partial F statistic comparing the fit of the current model to that on the line above. [f]: P-value of Partial F-test.

**(i) lm(nMT ~ MD *SLE)[a]**	**Df[b]**	**Sum Sq[c]**	**Mean Sq[d]**	**F[e]**	**P[f]**
MD	1	162.5	162.48	165.84	1.16×10^{-37}
SLE	1	2.6	2.57	2.63	0.11
qMD *SLE	1	3.4	3.40	3.47	0.062
Residuals	10462	10249.9	0.98		
**(ii) lm(nMT ~ SLE* MD)**	**Df**	**Sum Sq**	**Mean Sq**	**F**	**P**
SLE	1	17.5	17.48	17.84	2.42×10^{-5}
MD	1	147.6	147.57	150.63	2.18×10^{-34}
SLE* MD	1	3.4	3.40	3.47	0.062
Residuals	10462	10249.9	0.98		
**(iii) lm(nMT ~ MD *CSA)**	**Df**	**Sum Sq**	**Mean Sq**	**F**	**P**
MD	1	172.8	172.84	176.32	6.25×10^{-40}
CSA	1	4.9	4.93	5.03	0.025
MD *CSA	1	0.4	0.35	0.36	0.55
Residuals	11028	10810.7	0.98		
**(iv) lm(nMT ~ CSA* MD)**	**Df**	**Sum Sq**	**Mean Sq**	**F**	**P**
CSA	1	18.4	18.41	18.78	1.48×10^{-05}
MD	1	159.4	159.36	162.56	5.68×10^{-37}
SLE* MD	1	0.4	0.35	0.37	0.55
Residuals	11031	10810.7	0.98		

Table S5 related to Table 2: Effect of MD, number of stressful life events (SLE) and their interaction on normalised measure of mean telomere length (nTel). See **Table S4** for description.

**(i) Im(nTel ~ MD *SLE)**	**Df**	**Sum Sq**	**Mean Sq**	**F**	**P**
MD	1	60.9	60.88	62.38	3.11×10^{-15}
SLE	1	2.4	2.44	2.50	0.11
MD *SLE	1	0.3	0.29	0.30	0.59
Residuals	10685	10427.9	0.98		

**(ii) Im(nTel ~ SLE* MD)**	**Df**	**Sum Sq**	**Mean Sq**	**F**	**P**
SLE	1	9.7	9.67	9.91	0.0017
MD	1	53.7	53.65	54.97	1.31×10^{-13}
SLE* MD	1	0.3	0.29	0.30	0.59
Residuals	10685	10427.9	0.98		

**(iii) Im(nTel ~ MD *CSA)**	**Df**	**Sum Sq**	**Mean Sq**	**F**	**P**
MD	1	65.5	65.47	67.97	1.85×10^{-16}
CSA	1	0.5	0.53	0.55	0.46
MD *CSA	1	2.7	2.69	2.79	0.095
Residuals	11273	10859.0	0.96		

**(iv) Im(nTel ~ CSA* MD)**	**Df**	**Sum Sq**	**Mean Sq**	**F**	**P**
CSA	1	4.0	4.03	4.18	0.041
MD	1	62.0	61.97	64.33	1.16×10^{-15}
CSA * MD	1	2.7	2.69	2.79	0.095
Residuals	11273	10859.0	0.96		

Supplemental Experimental Procedures

Mitochondrial DNA and telomere length measures: relationship to cellular composition of saliva

We considered the possibility that the increased amounts of mtDNA in cases with MD might be due to systematic differences in the cellular composition of the saliva samples between cases and controls. If there were higher percentages of cell types that have more mtDNA in cases than from controls, the association between mtDNA amount and case status would reflect differences in the cellular composition of saliva between cases and controls.

We began by assessing the cellular composition of the saliva. Microscopy examination of the saliva samples revealed that the majority of cells were leucocytes, but there were not enough intact cells to obtain reliable estimates of the cellular composition; the samples were certainly not good enough for flow cytometry. Published methods obtain saliva after asking subjects to chew paraffin [S1], and while this generates a good flow of saliva, it would not be representative of how we collected DNA, and would introduce a confound that would vitiate extrapolating results to our sample. From the literature, in agreement with the observations we made, most of the cells in saliva are leukocytes, dominated by neutrophils (as in the blood) (see for example [S2-S5]). More recent analyses (e.g. [S1]) used cell sorting to show that the white cells are neutrophils and T-cells in approximately a 2:1 ratio, with smaller numbers of B-cells. We approached the problem of accounting for cellular admixture in the following ways (i) we used the methylation state of each sample to index cellular composition; (ii) we assessed the cellular composition of blood in stressed and non-stressed mice.

(i) Identification of cell composition of saliva samples using methylation patterns

The cellular origin of DNA can be identified from cell-type specific methylation marks that can be interrogated by sequencing after bisulfite conversion of unmethylated cytosine into thymine. 20 CpG loci can accurately predict the identity of leukocyte subsets and buccal epithelial: using this approach, estimates of the proportions of cell types are close to 100% accurate [S6]. We picked 13 CpG sites methylated in specific blood cell types that could best distinguish between B lymphocytes, T lymphocytes, Eosinophils, Basophils, NK cells, Monocytes, and Granulocytes and Neutrophils[6], and 7 CpG sites most significantly differently methylated in buccal epithelial cells and blood cells [S7, S8]. We designed primers for targeted sequencing of these CpG sites on 156 samples after bisulfite conversion of their saliva DNA using Sequenom's EpiDesigner BETA. We chose the 156 samples from the extremes of the mtDNA distribution, and matched for age. We constructed libraries using the WaferGen SmartChip MyDesign Target Enrichment system, and performed the targeted sequencing using Illumina Miseq. We cleaned the sequencing reads for adaptor sequences both at ends and in the middle of the reads before mapping them to the hg18 reference genome that has been processed to the bisulphite converted sequence using Bismark (v0.13.0) [S9] and excluding overlapping regions of paired-end reads to prevent double counting of methylated and unmethylated CpG sites. We then quantified the percentage methylation at all 20 CpG sites using Bismark's methylation extraction utility with the –bedGraph option.

Methylation states were readable at 19 sites for more than 90% of individuals. One site (cg14088196), an assay for epithelial cells, gave useable data for only 4% of individuals. Since six other epithelial states interrogated epithelial cells, the loss of this site does not invalidate our ability to quantitate epithelial cell composition. Figure S1 shows the percentage methylation at each of the 20 CpG sites in cases and controls. We proceeded to look for association between the molecular markers, amount of mtDNA and telomere length and the 19 methylation sites with high quality data. In assessing association between the methylation state and MD, we controlled for a number of potential confounds

including the age of samples. We quantile normalized the measures before using linear regression to determine association with amount of mtDNA and telomere length, and logistic regression with case-control status.

We determined the extent to which methylation sites account for the relationship between amount of mtDNA and telomere length and MD, using a partial F test to compare models. Methylation state significantly predicts case status (as expected from the differences between cases and controls shown also in Figure S1), but this association cannot explain the association between MD and amount of mtDNA, as shown by the non significant partial F-test ($P = 0.88$). There is a modest improvement in fit for telomere length, but not sufficient to account for the association between MD and telomere length.

To show the effect of taking into account methylation status in assessing the relationship between the molecular markers and MD, we calculated the residuals from models in which methylation marks predict amount of mtDNA and telomere length, and then compared the distributions of residuals to those of the original data in 156 samples. These results are shown in Figure S2 showing that the differences between cases and controls persist, once methylation states (cellular composition) is taken into consideration. We conclude that cellular composition cannot explain the difference in amount of mtDNA and mean telomere length between cases and controls.

(ii) Count of blood cells from stressed/non-stressed mice and qPCR quantification of mtDNA levels

We stressed 8 male and 8 female C57BL/6J mice for 4 weeks (using the protocol described in the Methods section), while maintaining 8 male and 8 female mice of the same inbred strain for 4 weeks without stress treatment. We measured blood mtDNA levels by qPCR and conducted blood cell counts for all male mice at 2 and 4 weeks after start of the experiment, and tested the effect of percentage of lymphocytes, neutrophils, monocytes and eosinophils (out of total white blood cell count) on mtDNA levels using linear regression after controlling for number

of weeks after start of experiment. We found no significant association between mtDNA levels and percentages of any leukocyte cell types, (ANOVA P values were 0.28, 0.30, 0.72, and 0.68 for lymphocytes, neutrophils, monocytes and eosinophils respectively) and the significant association between mtDNA levels with stress treatment cannot be explained by percentage of different leukocyte cell types. Similar results were found for an analysis of liver mtDNA levels and leukocyte cell types.

Regression analysis for causal effects between stress, MD and mtDNA and telomere length

We investigated whether the direction of causality in the relationship between stress, MD, and amount of mtDNA or mean telomeres length fitted either of the models:

stress -> MD -> mtDNA, telomeres (A)

stress -> mtDNA, telomeres -> MD (B)

stress -> mtDNA, telomeres, MD (C)

Model (C) corresponds to the situation that MD, amount of mtDNA and mean telomere length are conditionally independent after adjusting for stress. Since stress occurs earliest in time any causal model must have stress as the initial stimulus.

To obtain the proportion of variance in MD that is explained by different types of stress (which we subdivide into SLE: number of stressful life events and CSA: occurrence of childhood sexual abuse) and by amount of mtDNA or mean telomere length, we performed an analysis of deviance of the logistic regression models shown in Tables S2 and S3. All logistic regression models included as covariates the first three principal components (PCs) from a principal component analysis (PCA) performed with Genome-wide Complex Trait Analysis (GCTA) v1.24.4 [S10] using a genetic relationship matrix (GRM). The GRM was generated with 561,819 common, tagging SNPs from all autosomes. All SNPs in

this tagging set were polymorphic in 1000G Phase 1 ASN Panel, occur at greater than 5% minor allele frequency in CONVERGE study samples, and are out of linkage disequilibrium (LD) with each other (maximum pairwise LD = 0.8).

Both SLE and CSA have significant associations with MD risk regardless of conditioning on amount of mtDNA and telomere length. This suggests both SLE and CSA alter the risk to MD independently of amount of mtDNA and mean telomere length, or at least contain important additional information not captured by amount of mtDNA or telomere length. There is a significant but small interaction between SLE and amount of mtDNA in conferring risk to MD, but none between CSA and amount of mtDNA and none between telomere lengthand either SLE or CSA. This suggests that changes in amount of mtDNA may alter the risk to MD in presence of stress, though the variance in MD risk explained by the interaction is small.

Similarly, to investigate the dependence of amount of mtDNA and mean telomere length on stress and MD, we performed analysis of variance on the linear regression models shown in Tables S4 and S5. All linear regression models controlled for principal components 1 to 3 as above.

Both SLE and CSA have significant associations with amount of mtDNA and telomere length until conditioned upon MD, which also explains much greater variance in amount of mtDNA and telomere length than both SLE and CSA. No interactions between either stress measures and MD were found, suggesting their effect on amount of mtDNA and telomere length is largely mediated through MD, and MD does not modify the response in amount of mtDNA and telomere length to stress. From these analyses we conclude that the model (A) is the most consistent with the data.

Supplemental References

S1. Vidovic, A., Vidovic Juras, D., Vucicevic Boras, V., Lukac, J., Grubisic-Ilic, M., Rak, D., and Sabioncello, A. (2012). Determination of leucocyte subsets in human saliva by flow cytometry. Archives of oral biology *57*, 577-583.

S2. Wright, D.E. (1968). The differential leucocyte count of human saliva. Archives of oral biology *13*, 1159-1161.

S3. Schiott, C.R., and Loe, H. (1970). The origin and variation in number of leukocytes in the human saliva. Journal of periodontal research *5*, 36-41.

S4. Schiott, C.R., and Loe, H. (1969). The origin and variation in the number of leukocytes in the human saliva. Journal of periodontal research. Supplement, 24-26.

S5. Raeste, A.M. (1972). The differential count of oral leukocytes. Scandinavian journal of dental research *80*, 63-67.

S6. Accomando, W.P., Wiencke, J.K., Houseman, E.A., Nelson, H.H., and Kelsey, K.T. (2014). Quantitative reconstruction of leukocyte subsets using DNA methylation. Genome Biol *15*, R50.

S7. Slieker, R.C., Bos, S.D., Goeman, J.J., Bovee, J.V., Talens, R.P., van der Breggen, R., Suchiman, H.E., Lameijer, E.W., Putter, H., van den Akker, E.B., et al. (2013). Identification and systematic annotation of tissue-specific differentially methylated regions using the Illumina 450k array. Epigenetics & chromatin *6*, 26.

S8. van Dongen, J., Ehli, E.A., Slieker, R.C., Bartels, M., Weber, Z.M., Davies, G.E., Slagboom, P.E., Heijmans, B.T., and Boomsma, D.I. (2014). Epigenetic variation in monozygotic twins: a genome-wide analysis of DNA methylation in buccal cells. Genes *5*, 347-365.

S9. Krueger, F., and Andrews, S.R. (2011). Bismark: a flexible aligner and methylation caller for Bisulfite-Seq applications. Bioinformatics *27*, 1571-1572.

S10. Yang, J., Lee, S.H., Goddard, M.E., and Visscher, P.M. (2011). GCTA: a tool for genome-wide complex trait analysis. Am J Hum Genet *88*, 76-82.
